# Screening of *MAMLD1* Mutations in 70 Children with 46,XY DSD: Identification and Functional Analysis of Two New Mutations

**DOI:** 10.1371/journal.pone.0032505

**Published:** 2012-03-30

**Authors:** Nicolas Kalfa, Maki Fukami, Pascal Philibert, Francoise Audran, Catherine Pienkowski, Jacques Weill, Graziella Pinto, Sylvie Manouvrier, Michel Polak, Totsumo Ogata, Charles Sultan

**Affiliations:** 1 Service d'Hormonologie, Hôpital Lapeyronie, CHU de Montpellier et UM1, Montpellier, France; 2 Service de Chirurgie et Urologie Pédiatrique, Hôpital Lapeyronie, CHU de Montpellier et UM1, Montpellier, France; 3 Department of Molecular Endocrinology, National Research Institute for Child Health and Development, Tokyo, Japan; 4 Unité d'Endocrinologie Pédiatrique, Hôpital des Enfants, CHU de Toulouse, Toulouse, France; 5 Clinique de Pédiatrie, Hôpital Jeanne de Flandre, CHU de Lille, Lille, France; 6 Unité d'Endocrinologie Pédiatrique, Hôpital Necker Enfants Malades, APHP, Paris, France; 7 Service de Génétique Clinique, Hôpital Jeanne de Flandre, CHU de Lille, Lille, France; 8 Unité d'Endocrinologie et Gynécologie Pédiatriques, Service de Pédiatrie, Hôpital Arnaud de Villeneuve et UM1, CHU de Montpellier, Montpellier, France; Florida International University, United States of America

## Abstract

More than 50% of children with severe 46,XY disorders of sex development (DSD) do not have a definitive etiological diagnosis. Besides gonadal dysgenesis, defects in androgen biosynthesis, and abnormalities in androgen sensitivity, the Mastermind-like domain containing 1 (*MAMLD1*) gene, which was identified as critical for the development of male genitalia, may be implicated. The present study investigated whether *MAMLD1* is implicated in cases of severe 46,XY DSD and whether routine sequencing of *MAMLD1* should be performed in these patients.

Seventy children with severe non-syndromic 46,XY DSD of unknown etiology were studied. One hundred and fifty healthy individuals were included as controls. Direct sequencing of the *MAMLD1, AR, SRD5A2* and *NR5A1* genes was performed. The transactivation function of the variant *MAMLD1* proteins was quantified by the luciferase method.

Two new mutations were identified: p.S143X (c.428C>A) in a patient with scrotal hypospadias with microphallus and p.P384L (c.1151C>T) in a patient with penile hypospadias with microphallus. The *in vitro* functional study confirmed no residual transactivating function of the p.S143X mutant and a significantly reduced transactivation function of the p.P384L protein (*p* = 0.0032). The p.P359S, p.N662S and p.H347Q variants are also reported with particularly high frequency of the p.359T- p.662G haplotype in the DSD patients.

Severe undervirilization in XY newborns can reveal mutations of *MAMLD1. MAMLD1* should be routinely sequenced in these patients with otherwise normal *AR, SRD5A2* and *NR5A1*genes.

## Introduction

The disorders of sex development (DSD) comprise a variety of anomalies defined by congenital conditions in which chromosomal, gonadal, or anatomical sex is atypical. The prevalence of the 46,XY disorders of sex development (46,XY DSD) is difficult to determine with accuracy because of the heterogeneity in the clinical presentation and the etiologies. The estimated incidence of severe 46,XY DSD with uncertain sex is 2.2 per 10,000 births [1], and for a minor form of 46,XY DSD with isolated and non-severe hypospadias, the incidence is estimated at 1 in 250–400 births [2]. Two independent surveillance systems in the United States, the nationwide Birth Defects Monitoring Program (BDMP) and the Metropolitan Atlanta Congenital Defects Program (MACDP), reported a near doubling in the hypospadias rate in comparison with the immediately preceding decades [3]. Although recent studies have questioned this reported rise and provide conflicting data [4], [5], the elucidation of the pathophysiology of these genital malformations remains challenging.

The etiologies of 46,XY DSD are usually gonadal dysgenesis (defect in *SRY* and downstream genes such as *SOX9, WT1, NR5A1* [6], [7], etc.), defects in androgen biosynthesis and, more frequently, abnormalities in androgen sensitivity. Unfortunately, more than 50% of children with severe 46,XY DSD presenting with uncertain sex do not have a definitive clinical diagnosis [8]. For instance, an *AR* gene defect is identified in less than 10% of the cases [9].

In addition to these well classified causes, a recent candidate gene was identified as critical for the development of male genitalia: the Mastermind-like domain containing 1 (*MAMLD1*) gene (formerly *CXorf6*). This gene was discovered during studies to find the gene responsible for X-linked myotubular myopathy, *MTM1*, which maps to proximal Xq28 [10]: *MAMLD1* was observed to be deleted in patients with both the myopathy and external genital malformations [10], [11], [12]. Polymorphisms of *MAMLD1* have been reported in patients with isolated hypospadias, the less severe form of 46,XY DSD, but these variants usually do not affect the transactivation of the protein [13], [14]. Conversely, severe 46,XY DSD with uncertain sex has been sparsely studied. To date, only one study has focused on these patients: Fukami et al. identified three nonsense mutations in four individuals from a group of 166 patients [15]. The aim of the present study was to determine whether *MAMLD1* is frequently implicated in newborns and children with severe 46,XY DSD with uncertain sex and whether *MAMLD1* should be routinely sequenced in these patients.

## Materials and Methods

### Patients and controls

Two hundred and twenty individuals were included in this study. Seventy children presented with non-syndromic 46,XY DSD of unknown etiology. According to the Quigley classification [16], 8 patients exhibited a stage 2 phenotype; 32 patients, stage 3; 20 patients, stage 4; 5 patients, stage 5; and 5 patients, stage 6. One hundred and fifty healthy individuals were included as controls. Controls were chosen among patients without urinary, genital, or endocrine disease, or any other congenital malformation. For instance, patients with acute appendicitis or operated on for circumcision without phimosis were included. This study was approved by the Institutional Review Board (CPP-Montpellier, ID RCB No. 2008-A00781-54). Written consent was obtained from the parents, carers or guardians on behalf of the participating minors. When a mutation was identified, other family members were examined if possible. The patients and controls were Caucasian.

### DNA extraction

DNA was extracted from peripheral blood using a QIAamp DNA blood minikit (Qiagen, Courtaboeuf, France).

### Mutational analysis of MAMLD1

Direct sequencing of *MAMLD1* coding exons and their flanking splice sites was performed in all patients and controls using primers as previously described [17]. The 3730xl DNA Analyzer (Applied Biosystems, Foster City, CA, USA) was used. Sequencing reactions were repeated twice with at least two different PCR products. The DNA sequences were compared with the sequences of normal controls and the reference genomes from the ensembl.org database (Ensembl: ENSG00000013619) and the genebank database (MIM: 300120, NCBI Gene ID: 10046). It is notable that the number of the cDNA and amino acids has been changed recently because of the recognition of a novel *MAMLD1* start codon. This report describes *MAMLD1* cDNA and amino acids according to the new system.

### Molecular analysis of androgen sensitivity

A molecular analysis of the androgen receptor (*AR*) and 5 alpha reductase type 2 (*SRD5A2*) genes was performed in all patients. Exons 1–8 of the *AR* gene were amplified by PCR using sets of primers and reactions previously described [18]. Molecular analysis of the *SRD5A2* gene (exons 1–5) was performed as previously reported [19]. PCRs were verified for correct length on agarose gel, purified with Qiaquick PCR columns (Qiagen), and sequenced with the ABI Prism Big Dye terminator sequencing kit. *NR5A1*was sequenced in 46,XY DSD children with low plasma testosterone as previously published [6], [20].

### Homology study

Ensembl.org detected the putative homologs of the human *MAMLD1* gene and alignments were made with the ClustalW software at http://www.ebi.ac.uk/Tools/msa/clustalw2/.

### Structure prediction

The potential impact of variants was first predicted using X *in-silico* tools for secondary structure, tertiary structure and prediction of the consequences of amino acid changes.

The secondary structure for wildtype and variants was predicted using JPred software [21] (http://www.compbio.dundee.ac.uk/www-jpred/). The relative accessibility of amino acids was studied with Netsurf software [22] (http://www.cbs.dtu.dk/services/NetSurfP/). The three-dimensional structure was predicted by the Protein Homology/analogY Recognition Engine (PhyreEngine) from the Structural Bioinformatics Group, Imperial College, London, at http:www.sbg.bio.ic.ac.uk/phyrew/. This tool can detect remote homologous proteins with similar tertiary structures, based on multiple sequence profiles with structure-based profiles [23].

The functional consequences of amino acid changes were predicted using four algorithms. Polyphen (Harvard, USA) [24], [25], Panther [26], Sift (University of British Columbia) [27] and SNP-3D (University of Maryland) [28] were used, respectively, at http://genetics.bwh.harvard.edu/pph/, http://www.pantherdb.org/tools/csnpScoreForm.jsp., http://sift.jcvi.org/, and http://www.snps3d.org/modules.php?name=Search&op=advanced%20search. These algorithms are based on the alignment of orthologous and/or paralogous protein sequences and/or structural constraints.

### Transactivation analysis of MAMLD1

The transactivation function of the variant MAMLD1 proteins was analyzed by the luciferase method [29]. We used the previously reported luciferase reporter vector containing the promoter sequence of mouse hairy/enhancer of split 3 (*Hes3*) (–2,715∼+261 bp) [30] and expression vectors containing cDNAs for wildtype *MAMLD1*, p.S143X and p.P384L [29]. Mouse Leydig tumor (MLTC1) cells (ATCC, CRL-2065) seeded in 12-well dishes (0.5–1.0×10^5^ cells/well) were transiently transfected using Lipofectamine 2000 (Invitrogen) with 0.6 µg of luciferase reporter vector and 0.6 µg of expression vector for wildtype or variant *MAMLD1*, together with 20 ng of pRL-CMV vector (Promega) used as an internal control. As a control for the expression vectors, an empty counterpart vector was transfected. Luciferase assays performed with a Lumat LB9507 (Berthold) 48 hours after transfection were repeated three times.

### Statistical methods

Haplotype frequencies were compared between cases and controls using the χ^2^ test and the Fisher test on SPSS 16.0 software. The odds ratio (OR) was also considered with the logit confidence intervals method: 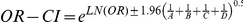. Hapmap and ensembl.org were used to exclude linkage disequilibrium. Regarding the transactivation analysis of *MAMLD1*, the results are expressed using the mean and SD, and statistical significance was determined by the *t*-test.

## Results

### Mutations of MAMLD1 and functional analyses

**Figure 1:**
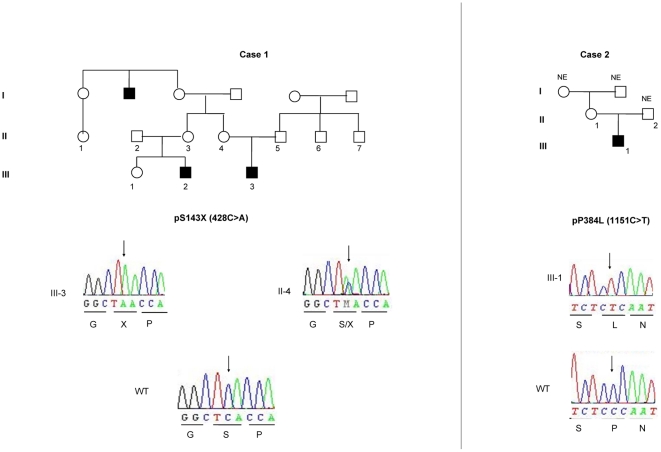
Electrochromatograms and pedigrees of the three patients with *MAMLD1* mutations. The black squares indicate patients with posterior hypospadias. All mutant sequences were controlled by wildtype (WT) DNA. Regarding case 1's family, only the members III-3 and II-4 were genotyped, as the other members in the pedigree declined genetic testing.

Among the 70 newborns and children with 46,XY DSD, two new mutations were identified in two unrelated patients: p.S143X (c.428C>A) and p.P384L (c.1151C>T) (Fig. 1). The clinical and genetic data are summarized in Table 1. None of these mutations was noted in the control group. The sequences of the *AR, SRD5A2* and *NR5A1* genes were normal in these patients.

**Table 1:** Clinical and hormonal data of patients with mutated *MAMLD1*.

Patient	Case 1	Case 2
*MAMLD1* mutation	pS143X	pP384L
Previous medical history	None	Maternal diabetes
Genital phenotype		
Urethral meatus	Scrotal	Penile posterior
Age at exam (yr,mo)	0,0	0,0
Microphallus	Yes, 20 mm	Yes, 20 mm with cuvature
Testis position	Intra-scrotal	Intra-scrotal
Testis size (normal = 1–2 ml)	Normal	Normal
Scrotal appearance	Ventral transposition, Bifid Scrotum	Bifid Scrotum
Renal and urinary tract structure	Normal	Normal
Extragenital phenotype	Normal	Normal
Growth		
Birth height, cm (SDS)	51 (+0)	50.5 (+0)
Birth weight, Kg (SDS)	3.540 (+0)	3.750 (+0.5)
Serum hormone level		
Time of measurment (yr,mo)	0,0	0,3
Testosterone (ng/ml) (1–3 ng/ml)	1.78	<0.07
LH (UI/l) (1–12 UI/l)	10	0.3
FSH (UI/l) (1–10 UI/l)	0.8	0.8
AMH	336 ng/ml	19 ng/ml*
Inhibin	NA	<15 ng/ml*

SD: standard deviation. ND: not determined. NA: not available. DHT: dihydrotestosterone. DHEA: dihydroepiandrsosterone. Parentheses indicate the standard deviation for height and weight and the normal range for hormone serum levels. Testes of 1–2 ml can be regarded as normal, as recently reported by Shibata et al. [34].

**Figure 2:**
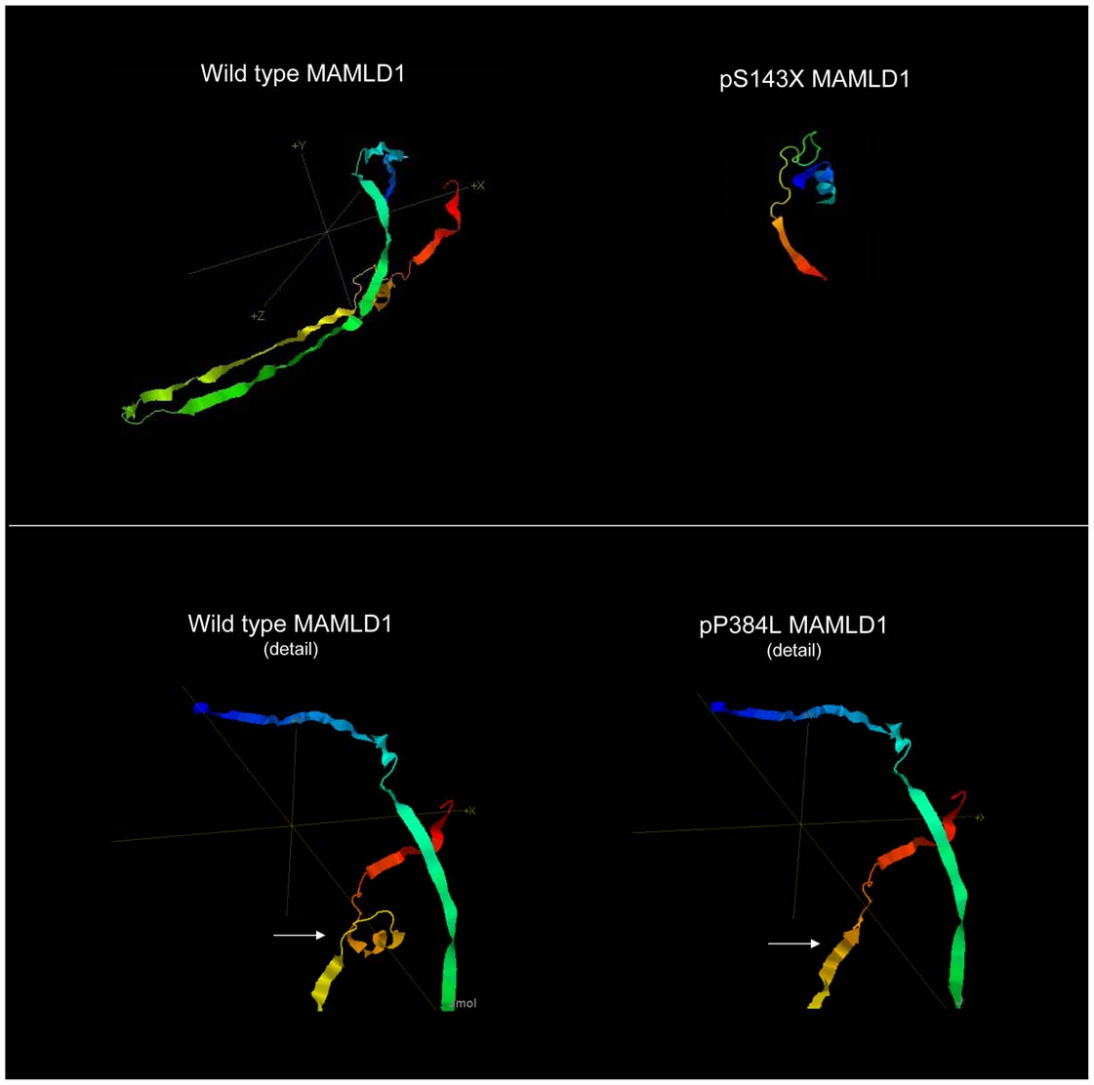
Tertiary structure prediction of the wildtype protein (left column) and with the mutants. 3D structure was predicted at Protein Homology/analogY Recognition Engine (PhyreEngine) from the Structural Bioinformatics Group, Imperial College, London, at http:www.sbg.bio.ic.ac.uk/phyre~/. The plain arrows show the changes in the shape of the protein between the wildtype and p.P384L.

**Figure 3:**
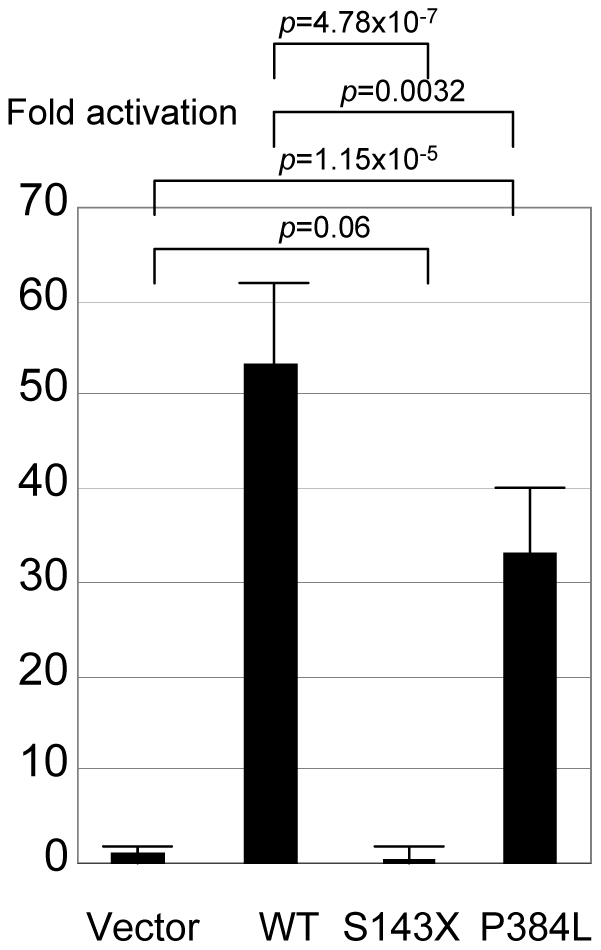
Transactivation function of the variants of the MAMLD1 protein analyzed by the luciferase method. The activity is evaluated for pHes3-luc vector.

* It is notable that anti-mullerian hormone and inhibin were lowered in one case. *MAMLD1* is indeed reported to be expressed in Sertoli cells, as well [15].

a- The p.S143X mutation was predicted to cause a short and truncated protein. The *in silico* prediction showed profoundly modified amino acid accessibility and 3D structure. Relative surface accessibility and absolute surface accessibility of the last amino acid changed from 0.248 to 0.834 and from 29.124 to 97.721, respectively. PhyreEngine predicted the loss of any functional site without a residual consensus sequence (no homologous sequence over 5% through whole genome) (Fig. 2). The *in vitro* functional study confirmed no residual transactivating function of the mutant (Fig. 3). Interestingly, a maternal uncle and a maternal cousin of the index case both exhibited severe hypospadias (not available for genetic testing). The mother was indeed heterozygous for the mutation (Fig. 1).

b- The p.P384L mutation was found in a patient with posterior penile hypospadias and microphallus. No cryptrochidism was noted. The secondary structure was predicted to be changed in the next four amino acids. The relative and absolute accessibilities of the amino acid were modified from 0.27 to 0.35 and from 39.07 to 65.25, respectively. The 3D structure prediction of the mutated protein was significantly changed (Fig. 2). All four *in silico* algorithms predicted affected protein function (Table 2) with a conserved amino acid throughout species (Table 3). Functional studies confirmed the significantly reduced transactivation function of the p.P384L protein with 60% residual activity when compared with the wildtype protein, *p* = 0.0032 (Fig. 3).

**Table 2:** Prediction of affected protein function using four algorithms.

Algorithm	pP384L
Polyphen	Probably damaging
	score = 0.961 (sensitivity: 0.71; specificity: 0.93)
Sift	Affect protein function
	Sift score = 0.04
Panther	Probability of deleterious effect = 0.42
	(subPSEC score = −2.7)
SNPS3D	Deleterious
	(svm score = −1.75)

References and online access are indicated in the text. Mathematical calculation of the significance of each score is available online.

**Table 3:** Homology study showed that this amino acid was highly conserved through species for the c.1041C>A and c.1151C>T mutations.

Patient	MSSNTLSGSTLRGS**L**NALLSSMTSSSNAAL
Human-MAMLD1	MSSNTLSGSTLRGS**P**NALLSSMTSSSNAAL
Pig	MSSSSLPGSTLHGS**P**GALLSSGAPSSSSAL
Horse	MSSSNLPGSTLQGS**P**NALLSSMVSGSSAAL
Chimpanzee	MSSNTLSGSTLRGS**P**NALLSSMTSSSNAAL
Mouse	MSSSSLSGSAVQSS**P**NALLSSMAPSSNASL
Rabbit	MAPHSLPGSSLQGS**P**NALLSSMAPNSSGAL
Dog	MASSNLPGSSFQAS**P**NALLASMASASSAGL
Cat	MASGNLPGSAFQGS**P**NALLASMASGSSAAL

### Polymorphisms of MAMLD1

We identified three polymorphisms of *MAMLD1* in our series: p.P359S (c.1075C>T, rs41313406), p.N662S (c.1985A>G, rs2073043) and p.H347Q (c.1041C>A, rs62641609). Regarding the p.P359S and p.N662S polymorphisms, 14 patients exhibited double polymorphisms (S-S haplotype) and five had the p.N359S polymorphism. The phenotypes of the patients with the S-S haplotype were as follows: penile posterior hypospadias and cryptorchidism in three cases, hypospadias and microphallus in five cases (anterior n = 1, penile posterior n = 2 and scrotal hypospadias n = 2), and cryptorchidism and microphallus in six cases (bilateral cryptorchidism n = 5, unilateral cryptorchidism n = 1). Using hapmap and ensembl.org, no linkage disequilibrium was found for these two variants. In previous studies, we and others found that the S-S haplotype was present in only 6/150 controls (4.0%) and 23/360 controls (6.4%) [13], [14]. By combining the published series for controls (matched patients and controls), we determined that the incidence of the S-S haplotype was higher in the DSD patients (20%, n = 70 vs. 6%, n = 510, *p* = 0.0003) (OR = 3.86, CI from 1.94 to 7.70, *p* = 0.05). Haplotypes and their relative frequencies in each group of patients are summarized in Table 4.

**Table 4:** Incidence of exonic polymorphisms p.P359S and p.N662S, and relative haplotypes in normal controls and 46,XY DSD patients.

Haplotype 359–662	Patients, n = 70	Controls, n = 510	Fisher, p value	OR	OR confidence interval (p = 0.05)
p.359C- p.662A	72.9% (n = 51)	90.6% (n = 462)	p = 0.0001	0.28	0.15–0.51
p.359T- p.662A	0%	1.5% (n = 8)	p = 0.60	0.42	0.02–7.35
p.359C- p.662G	7.1% (n = 5)	0.8% (n = 9)	p = 0.02	4.28	1.39–13.17
p.359T- p.662G (S-S polymorphism)	20% (n = 14)	6% (n = 31)	p = 0.0003	3.86	1.94–7.70

Controls are combined with the published series (matched for ethnicity of patients and controls) [13] [14]. The χ-square test was performed. When combining all patients with the p.662G polymorphism whatever the p.359 allele, this p.662G was significantly more frequent in 46,XY DSD patients: 27.1% (n = 19) vs. 6.8% (n = 40), *p* = 0.0001.

The p.H347Q variant, previously reported as a polymorphism especially in sub-Saharan populations (rs62641609, http://www.ensembl.org/Homo_sapiens/Variation/Summary?r=X:149638386-149639386v=rs62641609vdb=variationvf=16740729), was identified in a patient with posterior hypospadias and microphallus (25 mm length at birth).

## Discussion

*MAMLD1* is a good candidate to explore in patients with unexplained 46,XY DSD, as it has been shown to be expressed in fetal Leydig cells around the critical period for sex development [15]. The transient knockdown of *MAMLD1* mRNA expression results in significantly reduced testosterone production in mouse Leydig tumor cells [29]. *MAMLD1* is further coexpressed with steroidogenic factor (*NR5A1*), which regulates the transcription of genes involved in sex development, and an *NR5A1*target site was found within the *MAMLD1* gene [29], [31]. *MAMLD1* thus seems to have an important role in modulating testosterone production during sex development and is involved in the 46,XY disorders of sex development [32].

Regarding the minor forms of 46,XY DSD with isolated and non-severe hypospadias, mutational studies of *MAMLD1* have identified several polymorphisms in this gene. We reported the following variants in patients with isolated hypospadias: p.P359S, p.V505A, p.N662S and p.604ins3Q [13], [17], all of which were recently confirmed as polymorphisms [14]. The p.Q602K mutation was also found in one patient with posterior hypospadias and was predicted to affect the splicing process. An association between isolated hypospadias and the rare haplotype p.P359S-p.N662S is also suspected [13], [14].

Regarding severe 46,XY DSD with uncertain sex, only one published paper to date has reported three *MAMLD1* mutations (p.E124X, p.Q197X and p.R653X) [15]. It is precisely in this situation of severe genital malformation that the diagnosis of the causative mechanism is of clinical interest for medical treatment (hormone substitution, pubertal follow-up). In order to determine whether this report was an exceptional observation or of practical clinical interest, we screened 70 patients with severe 46,XY DSD of unknown origin. We identified two new mutations of *MAMLD1* in patients with severe hypospadias and microphallus (1 stop codon and 1 missense mutation). These mutations were associated with a severe phenotype, and reduced (p.P384L) or abolished (p.S143X) transactivation function was found in two cases. 46,XY DSD with normal *AR, SRD5A2* and *NR5A1*gene sequences can thus reveal a mutation of *MAMLD1*. This finding suggests a new diagnostic investigation for these patients and may be helpful in genetic counselling if a mutation is identified. It also provides new insight into the pathophysiology of DSD. Indeed, in the family of the child bearing the p.S143X mutation, the mother was heterozygous and two other males on the maternal side of the family exhibited a consistent phenotype. Unfortunately, the family declined any further investigation.

The mechanisms by which these mutations with reduced transactivation induce DSD are still under investigation. As noted above, several studies have provided strong evidence of *MAMLD1* implication in fetal sex development through modulation of testosterone production at the time of sex differentiation. The plasma testosterone measured in one of our cases was indeed lowered but it was normal in the other one, as previously reported in patients with nonsense mutations [15]. Plasma testosterone evaluation is thus not systematically helpful in orienting the diagnosis of DSD since mutations of the genes implicated in testosterone production - such as *MAMLD1* and *NR5A1* - have been reported in 46,XY DSD patients with normal plasma testosterone. These findings, along with the absence of correlation between the *in vitro* functional analysis and the biological and clinical phenotype, suggest that the genital malformation is primarily related to a transient prenatal testicular (Leydig cell) dysfunction and the resulting compromised testosterone production around the critical period of sex differentiation [33]. In the postnatal period, the mouse homolog of *MAMLD1* was indeed reported to be weakly expressed in the testis at one week of age and the expression was faint thereafter.

We also report a high incidence of the rare haplotype p.P359S-p.N662S in our series. The p.P359S (which was designated p.P286S in the previous report) variant was first reported in a patient with hypospadias but it was absent in his brother and nephew with the same phenotype [15]. The p.N662S (which was designated p.P589S in the previous report) variant was found in hypospadiac patients but was also reported in a normal population, although with low incidence [15]. We and others have found that the S-S haplotype is associated with a minor form of DSD, i.e., isolated hypospadias [14], but the *in vitro* functional study of the p.P359S-p.N662S *MAMLD1* variant was inconclusive with unchanged transactivation function [13]. In the present study, we show that the combination of these alleles was present in as much as 15% of patients with severe 46,XY DSD. This is significantly higher than in the controls [combining the series, 15% (n = 70) vs. 10.7% (n = 510), *p* = 0.0003]. Again, a transient testosterone production failure during prenatal development may have contributed to the undervirilization of the external genitalia, but how this haplotype can be present in normal, mild and severe phenotypes remains to be elucidated.

Severe undervirilization in XY newborns can reveal mutations of *MAMLD1*. *MAMLD1* should be routinely sequenced in these patients with otherwise normal *AR, SRD5A2* and *NR5A1* genes.
